# Utility of Whole Blood Thiamine Pyrophosphate Evaluation in *TPK1*-Related Diseases

**DOI:** 10.3390/jcm8070991

**Published:** 2019-07-08

**Authors:** Enrico Bugiardini, Simon Pope, René G. Feichtinger, Olivia V. Poole, Alan M. Pittman, Cathy E. Woodward, Simon Heales, Rosaline Quinlivan, Henry Houlden, Johannes A. Mayr, Michael G. Hanna, Robert D.S. Pitceathly

**Affiliations:** 1 MRC Centre for Neuromuscular Diseases, UCL Queen Square Institute of Neurology and National Hospital for Neurology and Neurosurgery, London WC1N 3BG, UK; 2 Department of Neuromuscular Diseases, UCL Queen Square Institute of Neurology, London WC1N 3BG, UK; 3 Neurometabolic Unit, National Hospital for Neurology and Neurosurgery, London WC1N 3BG, UK; 4 Department of Pediatrics, University Hospital Salzburg, Paracelsus Medical University, 5020 Salzburg, Austria; 5 Neurogenetics Unit, National Hospital for Neurology and Neurosurgery, London WC1N 3BG, UK; 6 Dubowitz Neuromuscular Centre, Great Ormond Street Hospital, London WC1N 3JH, UK

**Keywords:** mitochondrial diseases, *TPK1*, thiamine pyrophosphate, Leigh syndrome, thiamine deficiency

## Abstract

*TPK1* mutations are a rare, but potentially treatable, cause of thiamine deficiency. Diagnosis is challenging given the phenotypic overlap that exists with other metabolic and neurological disorders. We report a case of *TPK1*-related disease presenting with Leigh-like syndrome and review the diagnostic utility of thiamine pyrophosphate (TPP) blood measurement. The proband, a 35-year-old male, presented at four months of age with recurrent episodes of post-infectious encephalopathy. He subsequently developed epilepsy, learning difficulties, sensorineural hearing loss, spasticity, and dysphagia. There was a positive family history for Leigh syndrome in an older brother. Plasma lactate was elevated (3.51 mmol/L) and brain MRI showed bilateral basal ganglia hyperintensities, indicative of Leigh syndrome. Histochemical and spectrophotometric analysis of mitochondrial respiratory chain complexes I, II+III, and IV was normal. Genetic analysis of muscle mitochondrial DNA was negative. Whole exome sequencing of the proband confirmed compound heterozygous variants in *TPK1*: c. 426G>C (p. Leu142Phe) and c. 258+1G>A (p.?). Blood TPP levels were reduced, providing functional evidence for the deleterious effects of the variants. We highlight the clinical and bioinformatics challenges to diagnosing rare genetic disorders and the continued utility of biochemical analyses, despite major advances in DNA sequencing technology, when investigating novel, potentially disease-causing, genetic variants. Blood TPP measurement represents a fast and cost-effective diagnostic tool in *TPK1*-related diseases.

## 1. Introduction

Genetic disorders of thiamine transport and metabolism are a rare but treatable cause of thiamine deficiency that usually present during childhood [1]. Four genetic defects are reported; three present with a predominantly neurological phenotype (*SLC19A3*, *SLC25A19* and *TPK1*) and one with multisystem disease (*SLC19A2*), including megaloblastic anaemia, thrombocytopenia, diabetes, and hearing loss. Thiamine pyrophosphokinase 1 (hTPK1, EC2.7.6.2), encoded by *TPK1*, converts free thiamine to active thiamine pyrophosphate (TPP). Early recognition is therefore crucial given the potential benefits of thiamine supplementation. However, diagnosis of *TPK1*-related diseases is often delayed because of the clinical overlap with other metabolic diseases, including Leigh syndrome. Consequently, patients often undergo numerous investigations, including muscle and skin biopsies, prior to receiving a molecular diagnosis. Here, we describe a case of *TPK1*-related disease that highlights the potential applications of whole blood TPP measurement for diagnosis and subsequent biochemical monitoring following thiamine supplementation.

## 2. Case Presentation

**Figure 1:**
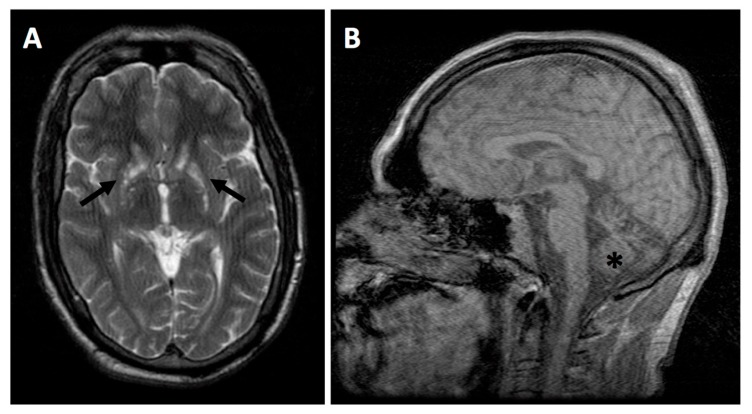
Brain MRI. (**A**) Axial image demonstrates bilateral, symmetrical T2-weighted high signal intensities within the corpus striatum (arrows). (**B**) Sagittal image shows cerebellar atrophy (asterisk).

The proband, the fourth child of healthy unrelated parents, was an uncomplicated pregnancy and delivery and reached all early motor milestones without delays. An older brother had been diagnosed with Leigh syndrome in the early childhood, while two older siblings were healthy. He presented with a post-viral encephalopathy aged four months following varicella-zoster infection and at nine months sensorineural hearing loss was detected. He subsequently experienced recurrent episodes of encephalopathy associated with intercurrent infection and was diagnosed with learning difficulties and seizures. At 22 years he was evaluated in a specialist mitochondrial clinic. At this time, he required fulltime use of a wheelchair and, despite improved seizure control with dual anticonvulsant therapy, continued to experience daily episodes of focal impaired awareness. Neurological examination revealed optic disc atrophy and sensorineural hearing loss. In limbs, there was a spastic increase in tone, hyper-reflexia, and bilateral extensor plantar responses. Laboratory tests, including CPK, fasting acylcarnitine profile, and very long chain fatty acids, were normal. Plasma lactate was elevated (3.51 mmol/L, reference range 0.5–2.22 mmol/L). Brain MRI revealed bilateral, symmetrical T2 hyperintensities in the corpus striatum and severe cerebellar and moderate brainstem volume loss, without signal abnormalities (Figure 1). The cervical spinal cord displayed normal imaging appearances.

**Figure 2:**
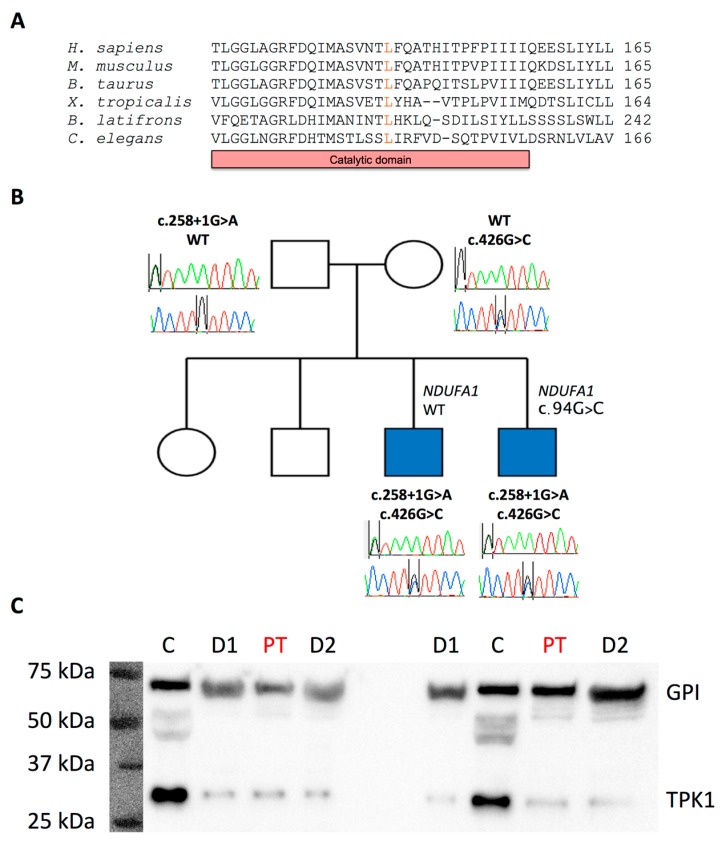
Domain and amino acid conservation for the c.426G>C; p.Leu142Phe variant, and segregation studies and impact on thiamine pyrophosphokinase 1 (TPK1) protein steady state levels of both *TPK1* variants. (**A**) Conservation of hTPK1 amino acid sequence between species. The L142 residue is highly conserved and resides within the catalytic domain of the protein. (**B**) Family pedigree and segregation analysis. *NDUFA1* variant: c.94G>C. *TPK1* variants (bold): c.426G>C and c.258+1G>A. (**C**) Steady state hTPK1 protein levels in patient fibroblasts. Abbreviations: GPI, glucose-6-phosphate isomerase (loading control); C, healthy control; D1 and D2, positive controls—previously reported as P2 and P5, respectively (Mayr et al., 2011) [6]; PT, proband in present study.

Pyruvate dehydrogenase complex (PDC) activity in cultured fibroblasts, and histological, histochemical, and mitochondrial respiratory chain enzyme analyses of skeletal muscle tissue, was normal. Sanger sequencing of the entire mitochondrial genome and molecular analysis for large scale mitochondrial DNA rearrangements using long range PCR were negative. Whole exome sequencing was performed using the proband’s genomic DNA extracted from peripheral leukocytes (Appendix A). Variants with a minor allele frequency (MAF) of ≥0.01 reported in gnomAD [2] and 1000 Genomes databases, and synonymous and deep intronic variants, were excluded. Recessive and X-linked inheritance patterns were prioritised (Supplementary Figure S1). First, a list of genes encoding mitochondrial-localized proteins (*n* = 1158, Mitocarta) [3] was analysed. A variant in *NDUFA1* (c.94G>C; p.Gly32Arg), previously reported in two cousins with a progressive neurodegenerative disorder associated with complex I deficiency [4], was identified. However, given the relatively high MAF reported in gnomAD (1189/205094, 408 hemizygotes, MAF 0.005797), and the normality of mitochondrial respiratory chain complex I enzyme activity in the proband’s muscle tissue, additional screening of variants detected in all remaining coding genes was undertaken. This revealed two missense mutations in *TPK1*: c.426G>C; p.Leu142Phe (MAF 0.000028 in gnomAD), which exists within a highly conserved region within the catalytic domain of hTPK1 (Figure 2A); and c.258+1G>A, residing in the donor splice site of intron 5 (Supplementary Figure S2). Segregation studies in affected and unaffected family members confirmed that both variants were in trans in the proband and his affected brother. Importantly, the *NDUFA1* variant failed to segregate with the disease (Figure 2B). TPP levels in the proband were low (35 nmol/L, reference range 67–265), providing biochemical support for the pathogenic effects of the variants, while hTPK1 steady state protein levels were reduced (Figure 2C), consistent with previous reports of *TPK1* mutations (see Appendix A for detailed methods) [5]. No additional bands were detected during the western blot analysis, indicating that the splicing variant most likely causes an unstable mRNA molecule. Oral thiamine, 100 mg twice a day, was commenced. Following the most recent assessment, TPP levels had increased to 59 nmol/L and lactate had normalized (1.74 mmol/L). However, no meaningful clinical improvement was observed.

## 3. Discussion

We report a new case of *TPK1*-related disease and highlight the clinical and diagnostic challenges of these potentially treatable disorders.

**Figure 3:**
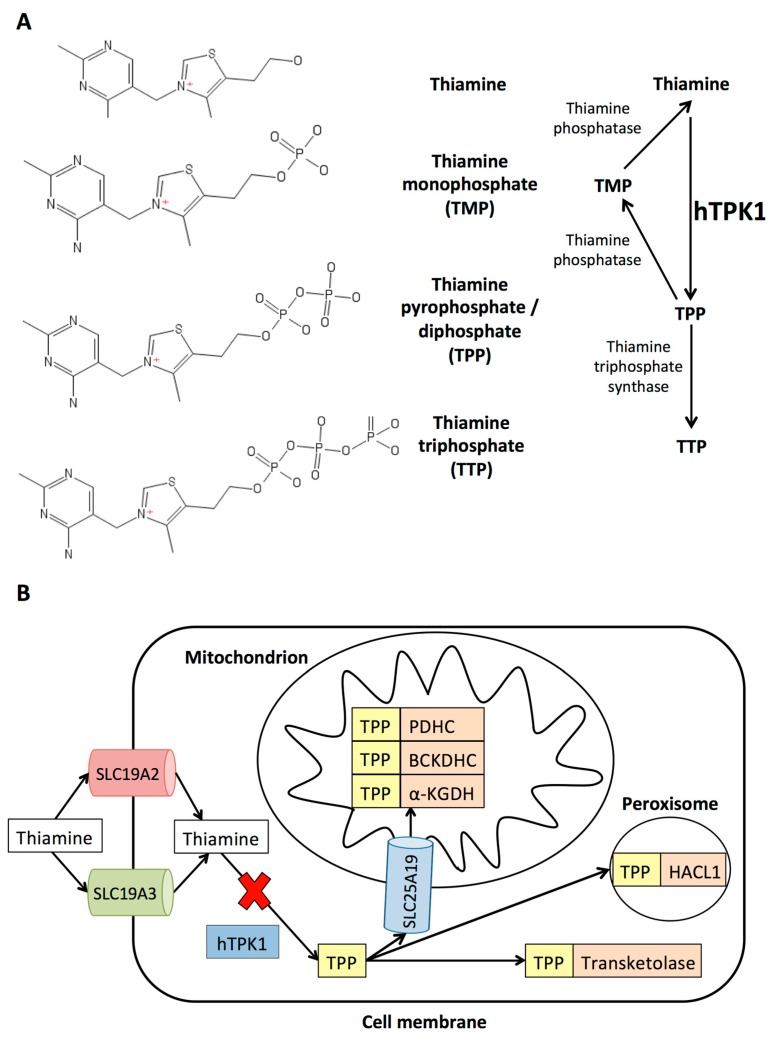
Thiamine structure and pathways. (**A**) Structure of different thiamine species and enzymatic pathways. Thiamine is phosphorylated to thiamine pyrophosphate (TPP) by thiamine pyrophosphokinase 1 (hTPK1). It can be further phosphorylated to thiamine triphosphate (TTP) by mitochondrial thiamine triphosphosphate synthase (ThTP synthase). It can also be dephosphorylated by thiamine phosphatase (Th phosphatase) to thiamine monophosphate (TMP) and thiamine. (**B**) Thiamine is absorbed in the small intestine and enters the cells using two transporters (encoded by *SLC19A2*/*SLC19A3*) where it is pyrophosphorylated to the active form (TPP) by hTPK1. TPP is transported into mitochondria by the *SLC25A19* encoded carrier where it acts as a cofactor for three distinct dehydrogenases: (1) pyruvate dehydrogenase complex (PDHC); (2) branched-chain alfa keto acid dehydrogenase (BCKDH); and (3) alpha-ketoglutarate dehydrogenase (α-KGDH). Outside of mitochondria, TPP acts a cofactor for Transketolase and 2-hydroxyacyl-Coa Lyase 1 (HACL1).

Inherited diseases of thiamine metabolism impair the transport and activation of thiamine. Thiamine exists in various phosphorylated states: unphosphorylated thiamine; thiamine monophosphate (TMP); thiamine diphosphate/pyrophosphate (TPP); and thiamine triphosphate (TTP) (Figure 3A). TPP is the active form of thiamine and acts as an important co-factor for several enzymes that play a major role in energy metabolism, including pyruvate dehydrogenase complex. Four primary genetic defects of thiamine metabolism are reported which impair different stages of thiamine transport or activation (Figure 3B). These include: (1) thiamine responsive megaloblastic anaemia (*SLC19A2*); (2) thiamine metabolism dysfunction syndrome 2 (biotin- or thiamine-responsive encephalopathy type 2, *SLC19A3*); (3) thiamine metabolism dysfunction syndrome 5 (episodic encephalopathy type, *TPK1*); and (4) thiamine metabolism dysfunction syndrome 4 (progressive polyneuropathy type, *SLC25A19*). 

*SLC19A3*-, *TPK1*-, and *SLC25A19*-related disorders present primarily with nervous system phenotypes that are difficult to distinguish from isolated mitochondrial encephalopathy. However, early recognition is important so that treatment with thiamine can be initiated. To facilitate this process, diagnostic criteria have recently been established [5]. Unfortunately, given their rarity, only limited data concerning *TPK1*-related diseases exists; only 13 patients are reported [5,6,7,8,9,10]. The majority of cases exhibited early onset, recurrent encephalopathic episodes, frequently triggered by infection, followed by ataxia, epilepsy, pyramidal signs, and dystonia. One family presented with progressive dystonia without recurrent encephalopathic episodes [10]. The clinical course of the disease tends to be severe, with high levels of physical disability and reduced lifespan; of the 13 patients, three died at 29 months, three, and eight years. There is no clear genotype–phenotype correlation and the response to thiamine supplementation is variable [5].

Our case highlights the diagnostic challenges in *TPK1*-related diseases. The clinical phenotype was compatible with Leigh syndrome, prompting investigations to exclude a mitochondrial disorder. However, muscle histochemistry and respiratory chain enzyme activity were normal and consistent with previous reports of TPK1-related disease. An unbiased next generation sequencing (NGS) approach helped identify two missense *TPK1* variants: c.426G>C (p.Leu142Phe) and c.258+1G>A (p.?). The c.426G>C variant has been reported once in ClinVar and considered as a likely pathogenic (RCV000255057). Interestingly, the c.425T>C variant affecting the same amino acid (p.Leu142Ser) has also been reported in ClinVar (RCV000650011). However, no further clinical information is reported. Given the molecular heterogeneity associated with Leigh syndrome, additional disease-causing candidate genes were also considered. The *NDUFA1* variant c.94G>C had been reported in two patients; one with a progressive neurodegenerative disease associated with complex I deficiency, and one with muscular hypotonia and lactic acidosis [4,11]. This association was supported by functional data in hamster cell lines confirming reduced complex I activity [4]. Nevertheless, segregation studies and analysis of recent MAF data among the general population suggests a non-pathogenic basis for the variant [12]. The *TPK1* variants were therefore considered the most likely cause of disease in our family. The pathogenic effects were supported by segregation studies and a confirmed reduction in both plasma TPP concentration and hTPK1 steady state protein levels. Thus, despite significant advances in genomics, ancillary biochemical, and protein studies remain an important tool when investigating rare genetic diseases.

**Figure 4:**
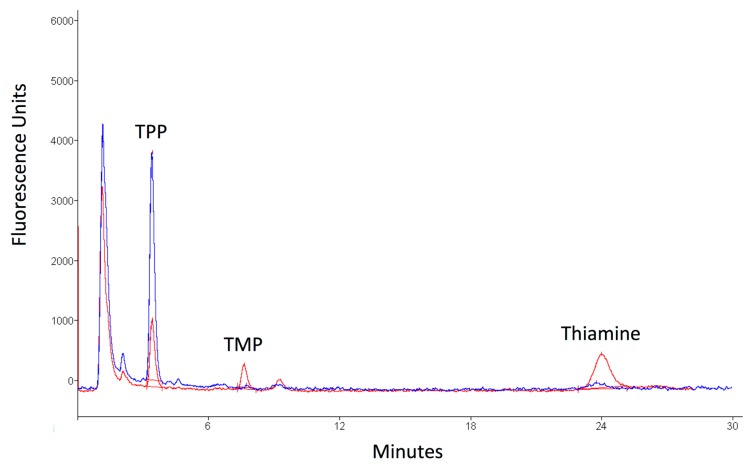
High performance liquid chromatography (HPLC) after 24 months of supplementation with thiamine. Red line: patient. Blue line: control. In patient there is a prominent peak for thiamine monophosphate (TMP) and unphosphorylated thiamine (thiamine) compared to control. These are caused by a reduced conversion of thiamine and TMP to thiamine diphosphate/pyrophosphate (TPP) for a deficient hTPK1 activity.

Despite the molecular heterogeneity exhibited by Leigh syndrome, TPP measurement represents a cost and time effective strategy when investigating suspected *TPK1*-related disease, with a high level of sensitivity; reported patients harbouring *TPK1* mutations in whom whole blood or plasma TPP was measured (7/13) exhibited reduced concentrations (Table 1). There are a number of analytical methods for Vitamin B1 measurement described in the literature, with high performance liquid chromatography (HPLC) and fluorescence detection of oxidised thiamine derivatives being the most commonly used [13,14]. The availability of numerous laboratory techniques to measure TPP levels, in addition to variations in sample size, demographics, and dietary intake of control samples, has potentially contributed towards observed differences in reported TPP reference ranges, with the bottom end being between 63 and 105 nmol/L [15]. Regardless of the methodology, the most common derivative measured in blood is TPP given that this is the most abundant form of Vitamin B1 present. However, additional phosphorylated and unphosphorylated forms of thiamine are observed using HPLC chromatograms and can be identified with reference to standards, albeit at low abundance, and sometimes at the limit of quantitation [13,16]. Recently described methods [16,17,18], using HPLC with fluorescence or mass spectrometric detection, can quantify other forms of thiamine. These methods have potential utility during the investigation of inborn errors of thiamine metabolism using biological samples where other thiamine species are more abundant. For example, in cerebrospinal fluid, thiamine and TMP are more abundant than TPP. In our patient, a commercially available vitamin B1 HPLC kit (Chromsystems Instruments and Chemicals GmbH, Munich, Germany) and an in-house derived reference range (67–265 nmol/L) was used to quantify TPP. The Chromsystems method extracts and derivatises vitamin B1 species from whole blood and then separates and detects these species by isocratic HPLC with fluorescence detection (https://www.chromsystems.com). Although this method is not designed to measure other forms of Vitamin B1, these can be observed as extra peaks in the chromatograms. This is particularly useful once the patient has commenced thiamine supplementation as prominent thiamine and TMP peaks are observed (Figure 4).

## 4. Conclusions

In conclusion, *TPK1*-related diseases pose a significant diagnostic challenge, given their clinical heterogeneity and overlap with other inherited metabolic and mitochondrial disorders. However, recognition is crucial to enable early therapeutic intervention with thiamine. TPP blood measurement represents a fast and cost-effective screening tool for suspected *TPK1*-related disorders to direct genetic testing and help filter potentially pathogenic variants generated by high-throughput sequencing methods. Research is ongoing to investigate our cohorts of children and adults with confirmed inborn errors of metabolism to evaluate the sensitivity and specificity of TPP measurement in this population.

## Figures and Tables

**Table 1:** Molecular and functional characteristics of reported *TPK1* mutations.

ID	*TPK1* Mutations	Predicted Effect on Protein	TPP Levels	Western Blot	Reference
P1	c.[148A>C];[501+4A>T]	p.[Asn50His];[Val119_Pro167del]	N/A	N/A	Mayr et al. [6]
P2	c.[148A>C];[501+4A>T]	p.[Asn50His];[Val119_Pro167del]	68 * (B)	↓hTPK1	Mayr et al. [6]
P3	c.[119T>C];[119T>C]	p.[Leu40Pro)];[Leu40Pro]	50.4 * (B)	↓hTPK1	Mayr et al. [6]
P4	c.[119T>C];[119T>C]	p.[Leu40Pro)];[(Leu40Pro]	N/A	N/A	Mayr et al. [6]
P5	c.[179_182delGAGA];[656A>G]	p.[Arg60LysfsTer52];[Asn219Ser]	96.9 * (B)	↓hTPK1	Mayr et al. [6]
P1 (P76 in [5])	c.[604T>G];[604T>G]	p.[Trp202Gly];[Trp202Gly]	N/A	N/A	Fraser et al. [8]
P2 (P77 in [5])	c.[604T>G];[604T>G]	p.[Trp202Gly];[Trp202Gly]	N/A	N/A	Fraser et al. [8]
P1 (P79 in [5])	c.[479C>T];[479C>T]	p.[Ser160Leu];[Ser160Leu]	60.9 ^ (B)	Normal	Banka et al. [7]
P2	c.[664G>C];[664G>C]	p.[Asp222His];[Asp222His]	85.4 ^ (B)	↓hTPK1	Banka et al. [7]
N/A	c.[656A>G];deletion of exons 3 and 4 on mRNA studies	p.[Asn219Ser];[?]	N/A	N/A	Invernizzi et al. [9]
Proband	c.[119T>C];[119T>C]	p.[Leu40Pro];[Leu40Pro]	32 ~ (P)	N/A	Mahajan et al. [10]
Sister	c.[119T>C];[119T>C]	p.[Leu40Pro];[Leu40Pro]	N/A	N/A	Mahajan et al. [10]
P78	c.[365T>C];[365T>C]	p.[Ile122Thr];[Ile122Thr]	N/A	N/A	Ortigoza-Escobar et al. [5]
Proband	c.[258+1G>A];[426G>C]	p.[Leu142Phe];[?]	35 ^†^ (B)	↓hTPK1	Present study
Brother	c.[258+1G>A];[426G>C]	p.[Leu142Phe];[?]	N/A	N/A	Present study

Abbreviations: B, Blood; N/A, Not Available; P, Plasma; TPP, thiamine pyrophosphate. * Mayr et al. TPP reference range 132–271 nmol/L; ^ Banka et al. TPP reference range 132.2–271 nmol/L; ~ Mahajan et al. TPP reference range 38–122 ng/L; † Present study TPP reference range 67–265 nmol/L.

## Supplementary Materials

The materials are available online at https://www.mdpi.com/2077-0383/8/7/991/s1.

## Appendix A

The study was performed under the ethical guidelines issued by our institution, with written informed consent obtained from participants for genetic studies.

### Whole Exome Sequencing

Genomic DNA was extracted from peripheral leukocytes and prepared using Agilent SureSelect Target Enrichment Kit preparation guide and sequenced on HiSeq 2000/2500 sequencer (Illumina). The Illumina fastq sequencing data was mapped to the human reference assembly, hg19 (GRCh37; UCSC genome browser), and variants were called using GATK. Filtering of variants was performed using VarAft platform [19]. Variants with a MAF of ≥0.01 in gnomAD, and 1000 Genomes databases and synonymous and deep intronic variants were excluded from further evaluation. Variants within genes encoding mitochondrial-localized proteins (*n* = 1158, Mitocarta) were first considered. If no candidate mitochondrial disease-related genes were identified, variants in all coding genes were analyzed.

### Western Blot Analysis

For western blot analysis, 10,000× *g* supernatants of fibroblast homogenates were used. Proteins were separated on a 10% acrylamide/bisacrylamide gel and transferred to nitrocellulose membranes (Biorad, Trans-Blot^®^ Turbo™ Mini Nitrocellulose Transfer Packs, #1704158) using the Transblot Turbo System (Biorad). The membranes were washed in Tris-buffered saline (TBS) for 5 min, air-dried for 30 min, then blocked for 30 min at room temperature in Western Blocking Reagent (Roche, #11921673001), and dissolved in TBS-T (0.5% Tween-20). After washing with TBS-T, the membranes were incubated with the primary antibody diluted in 1x Western Blocking Reagent Solution in TBS-T. The western blot was incubated with the following antibodies: polyclonal rabbit anti-TPK1 (Sigma-Aldrich, HPA021849, overnight at 4 °C, 1:1000) and polyclonal rabbit anti-glucose-6-phosphate isomerase (Santa Cruz, sc-33777, 1 h, room temperature, 1:1000). After washing with TBS-T, the membranes were incubated with the secondary antibody (Dako, Dako EnVision+ System-HRP Labelled Polymer Anti-Rabbit, K4003, 1 h, room temperature, 1:100 in 1× Western Blocking Reagent Solution in TBS-T). For horse-radish peroxidase (HRP), detection the Lumi-Light^PLUS^ Western Blotting Substrate (Roche, #12015196001) was used.
