# Supplementary material for: Utility of Whole Blood Thiamine Pyrophosphate Evaluation in TPK1-Related Diseases
Source: J Clin Med. 2019 Jul 8;8(7):991. doi: 10.3390/jcm8070991 (PMC6679130; doi:10.3390/jcm8070991)
Supplement: Supplementary file 1 [file jcm-08-00991-s001.zip › Supplementary Figure 1.doc]

**Supplementary Figure 1.** Filtering strategy applied to whole exome sequencing data. First we excluded all synonymous variants, deep intronic variants and variants with a minor allele frequency (MAF) >1%. We then examined variants in genes listed in Mitocarta and applied a Recessive and X-linked disease model. Two variants on Chromosome X were identified: *NDUFA1* c.94G>C:p.G32R (MAF: 0.0057); and *ACOT9* c.593A>G:p.H198R (MAF: 0.003). Given the relatively high MAF of both variants, we extended the analysis to include all coding regions within the exome. Further manual searching based on phenotype identified two candidate *TPK1* variants (c. 426G>C and c. 258+1G>A) that warranted further investigations.
