# Supplementary material for: Utility of Whole Blood Thiamine Pyrophosphate Evaluation in TPK1-Related Diseases
Source: J Clin Med. 2019 Jul 8;8(7):991. doi: 10.3390/jcm8070991 (PMC6679130; doi:10.3390/jcm8070991)
Supplement: Supplementary file 1 [file jcm-08-00991-s001.zip › Supplementary Figure 2.doc]

**Supplementary Figure 2.** Phylogenetic conservation of the donor splice site c.258+1G>A of exon 5 (GenBank NM_022445.4). The affected position is part of the canonical splice donor site. Splicing of this exon of TPK1 is conserved within vertebrates as is the guanosine. The following sequences (GenBank accession number) have been aligned by ClustalOmega (https://www.ebi.ac.uk/Tools/msa/clustalo/): Homo sapiens, human (NG_032112.2), Pan troglodytes, chimpanzee (NC_036886.1), Bos taurus, cattle (NC_037331.1: range 108101142-107671402), Rattus norvegicus, rat (NC_005103.4: range 72792252-73174179), Columba livia, pigeon (NW_004973403.1), Alligator mississippiensis, American alligator (NW_017708624), Gekko japonicus, Japanese gecko (NW_015165868.1), Latimeria chalumnae, West Indian Ocean coelacanth (NW_005819738.1), Danio rerio, zebrafish (NC_007135.7: range 18286427-18444729), Austrofundulus limnaeus, killifish (NW_013952452.1), Callorhinchus milii, Australian ghostshark (NW_006890243.1: range 708316-844463).
